# Supplementary material for: Time-resolved in vivo ubiquitinome profiling by DIA-MS reveals USP7 targets on a proteome-wide scale
Source: Nat Commun. 2021 Sep 13;12:5399. doi: 10.1038/s41467-021-25454-1 (PMC8438043; doi:10.1038/s41467-021-25454-1)
Supplement: Supplementary file 1 — Supplementary Information [file 41467_2021_25454_MOESM1_ESM.pdf]

Supplementary Information for:

Time-resolved in vivo ubiquitinome profiling by DIA-MS reveals USP7 targets on a proteome-wide scale

Martin Steger[1,*], Vadim Demichev[2,3,4,*], Mattias Backman[1], Uli Ohmayer[1], Phillip Ihmor[1], Stefan Müller[1], Markus Ralser[2,3] and Henrik Daub[1]

[1]Evotec München GmbH, Am Klopferspitz 19a, Martinsried, Germany

[2]Charité – Universitätsmedizin Berlin, Department of Biochemistry, Berlin, Germany

[3]The Francis Crick Institute, Molecular Biology of Metabolism Laboratory, London, UK

[4]The University of Cambridge, Department of Biochemistry and Cambridge Centre for Proteomics, Cambridge, UK

*equal contribution

Address correspondence to: martin.steger@evotec.com

Content

Supplementary Fig. 1: Benchmarking of the SDC lysis protocol.

Supplementary Fig. 2: Identification of K-GG peptides and proteins in the FT671 time course experiment.

Supplementary Fig. 3: USP7-dependent changes in the ubiquitinome and the proteome.

Supplementary Fig. 4: Modulation of protein ubiquitination and protein stability upon USP7 inhibition.

Supplementary Fig. 5: siRNA-mediated knockdown of USP7 in HCT116 cells.

Supplementary Fig. 6: FT671 time course experiment with short treatment times.

Supplementary Fig. 7: Detection of USP7 ubiquitination using ubiquitin binding domains.

Supplementary Fig. 8: PTM scoring module as implemented in DIA-NN.

Supplementary Fig. 9: Setup for library-free and spectral library-based searches in DIA-NN.

Supplementary Fig. 10: Identification confidence for K-GG peptides with DDA and DIA.

Supplementary Fig. 11: Benchmarking DIA ubiquitinomics.

Supplementary Note 1: Identification confidence for K-GG peptides with DDA and DIA

Supplementary Note 2: Quantification accuracy and precision for ubiquitinomics with DDA and DIA

Supplementary References

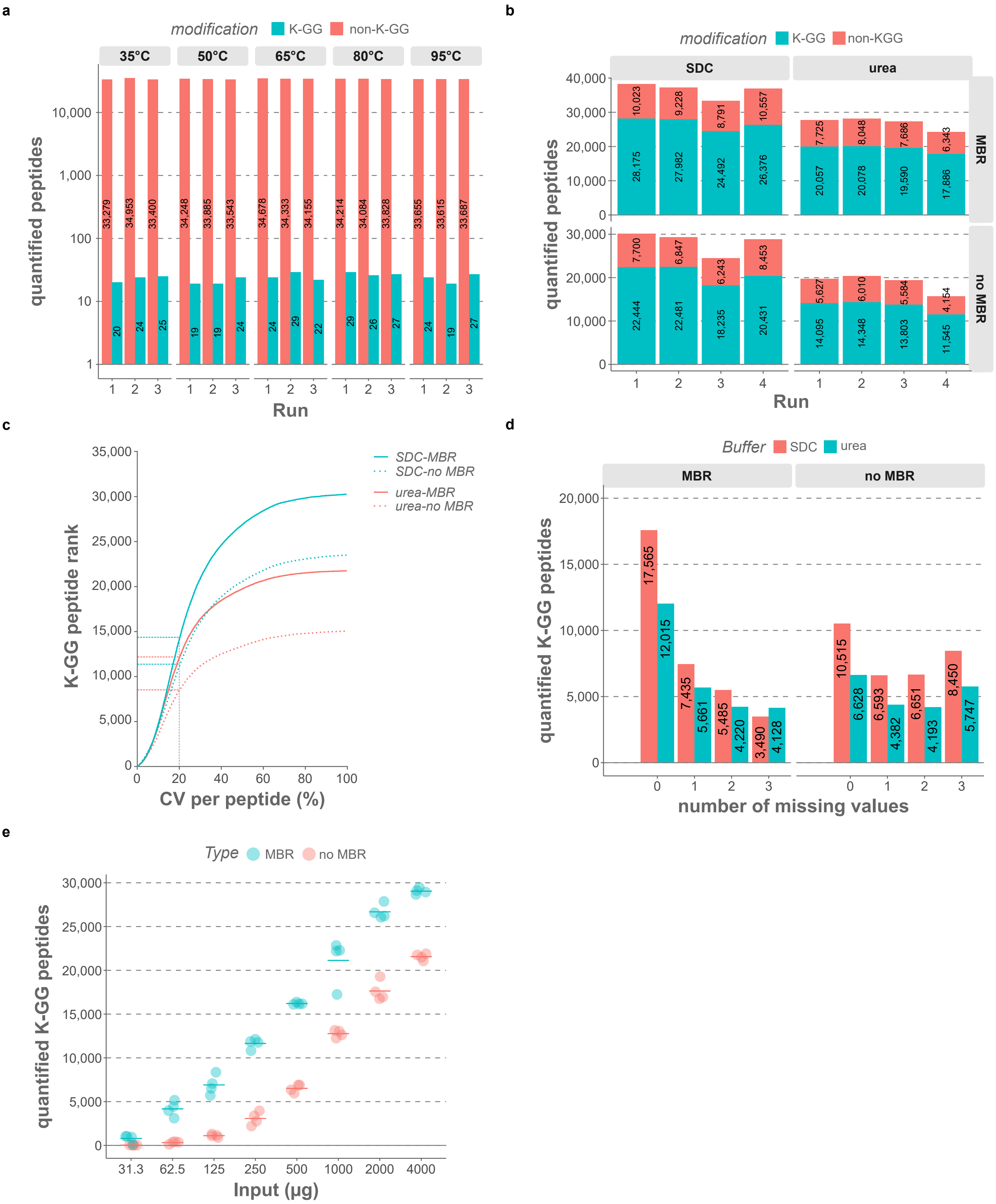
a
modification K-GG non-K-GG
35°C 50°C 65°C 80°C 95°C
quantified peptides
10,000
1,000
100
10
1
33,279 34,953 33,400 34,248 33,885 33,543 34,678 34,333 34,155 34,214 34,084 33,828 33,655 33,615 33,687
20 24 25 19 19 24 24 29 22 29 26 27 24 19 27
1 2 3
Run
b
modification K-GG non-KGG
SDC urea
MBR
no MBR
40,000
30,000
20,000
10,000
0
quantified peptides
10,023 9,228 8,791 10,557
28,175 27,982 24,492 26,376
7,725 8,048 7,686 6,343
20,057 20,078 19,590 17,886
7,700 6,847 6,243 8,453
22,444 22,481 18,235 20,431
5,627 6,010 5,584 4,154
14,095 14,348 13,803 11,545
1 2 3 4
Run
c
SDC-MBR
SDC-no MBR
urea-MBR
urea-no MBR
K-GG peptide rank
35,000
30,000
25,000
20,000
15,000
10,000
5,000
0
0 20 40 60 80 100
CV per peptide (%)
d
Buffer SDC urea
MBR no MBR
20,000
15,000
10,000
5,000
0
quantified K-GG peptides
17,565 12,015 7,435 5,661 5,485 4,220 3,490 4,128
10,515 6,628 6,593 4,382 6,651 4,193 8,450 5,747
0 1 2 3
number of missing values
e
Type MBR no MBR
quantified K-GG peptides
30,000
25,000
20,000
15,000
10,000
5,000
0
31.3 62.5 125 250 500 1000 2000 4000
Input (µg)

Supplementary Figure 1. Benchmarking of the SDC lysis protocol.

a HCT116 cells were lysed in SDC buffer without chloroacetamide (CAA), followed by incubation with 40 mM CAA at different temperatures (35/50/65/80/95°C) for 10 minutes. After tryptic digestion, the proteome was acquired in data-dependent acquisition mode (DDA) using a 75 min chromatographic gradient. The raw data were processed with MaxQuant and K-GG was included as a variable modification in the search. Plotted are K-GG and non-K-GG peptides for all replicates and conditions. **b** Fraction of unmodified and K-GG-modified peptides quantified from urea and SDC lysates in MG-132-treated HCT116 cells. The data were acquired using a 125 min LC gradient and the raw data were processed with MaxQuant, with or without match between runs (MBR). **c** Ranked K-GG peptide coefficients of variation (CVs) for SDC- and urea-based lysis protocols with (solid lines) or without (dashed lines) MaxQuant's MBR function enabled. The 20% CV cut-off is marked. **d** Number of K-GG peptide identifications with 0, 1, 2, or 3 missing values in quadruple measurement for SDC- (pink) and urea-based (turquoise) lysis methods. **e** Number of MS-quantified, ubiquitinated peptides (K-GG remnants) with different protein inputs in MG-132-treated Jurkat cells (6 h treatment). Four individual samples were processed for each condition and the data processed with or without MBR. Source data are provided as a Source Data file.

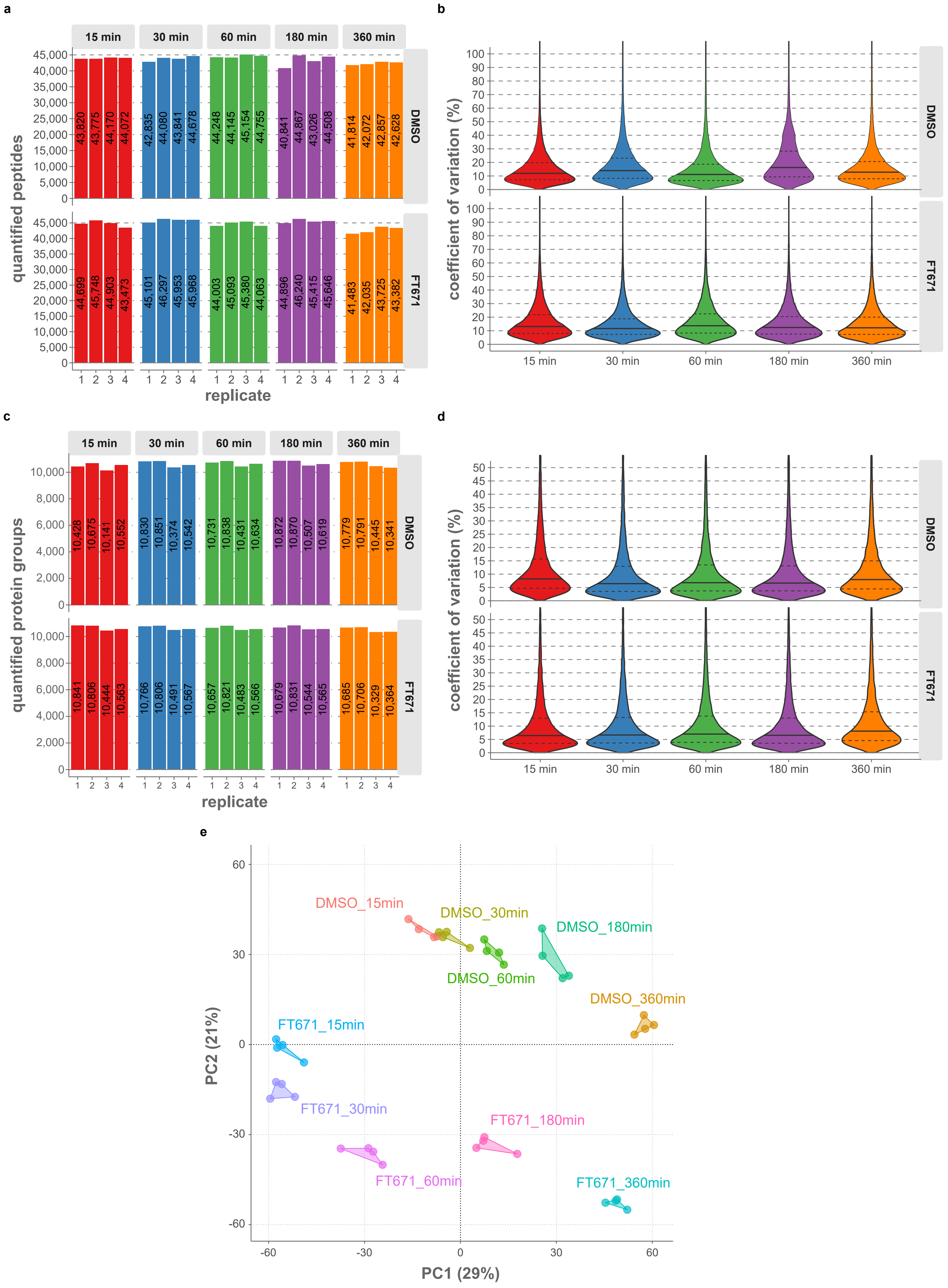
a
15 min
30 min
60 min
180 min
360 min
DMSO
FT671
quantified peptides
replicate
43,820
43,775
44,170
44,072
42,835
44,080
43,841
44,678
44,248
44,145
45,154
44,755
40,841
44,867
43,026
44,508
41,814
42,072
42,857
42,628
44,699
45,748
44,903
43,473
45,101
46,297
45,953
45,968
44,003
45,093
45,380
44,063
44,896
46,240
45,415
45,646
41,483
42,035
43,725
43,382
b
coefficient of variation (%)
DMSO
FT671
15 min
30 min
60 min
180 min
360 min
c
15 min
30 min
60 min
180 min
360 min
DMSO
FT671
quantified protein groups
replicate
10,428
10,675
10,141
10,552
10,830
10,851
10,374
10,542
10,731
10,838
10,431
10,634
10,872
10,870
10,507
10,619
10,779
10,791
10,445
10,341
10,841
10,806
10,444
10,563
10,766
10,806
10,491
10,567
10,657
10,821
10,483
10,566
10,679
10,831
10,544
10,565
10,685
10,706
10,329
10,364
d
coefficient of variation (%)
DMSO
FT671
15 min
30 min
60 min
180 min
360 min
e
DMSO_15min
DMSO_30min
DMSO_180min
DMSO_60min
DMSO_360min
FT671_15min
FT671_30min
FT671_180min
FT671_60min
FT671_360min
PC2 (21%)
PC1 (29%)

Supplementary Figure 2. Identification of K-GG peptides and proteins in the FT671 time course experiment.

a Number of MS-quantified K-GG peptides and **c** protein groups, with their coefficients of variation (CVs, in **b** and **d**, for ubiquitinome and proteome, respectively) for all replicates and treatment conditions. **e** Principal component analysis (PCA) of the ubiquitinomics data. Source data are provided as a Source Data file.

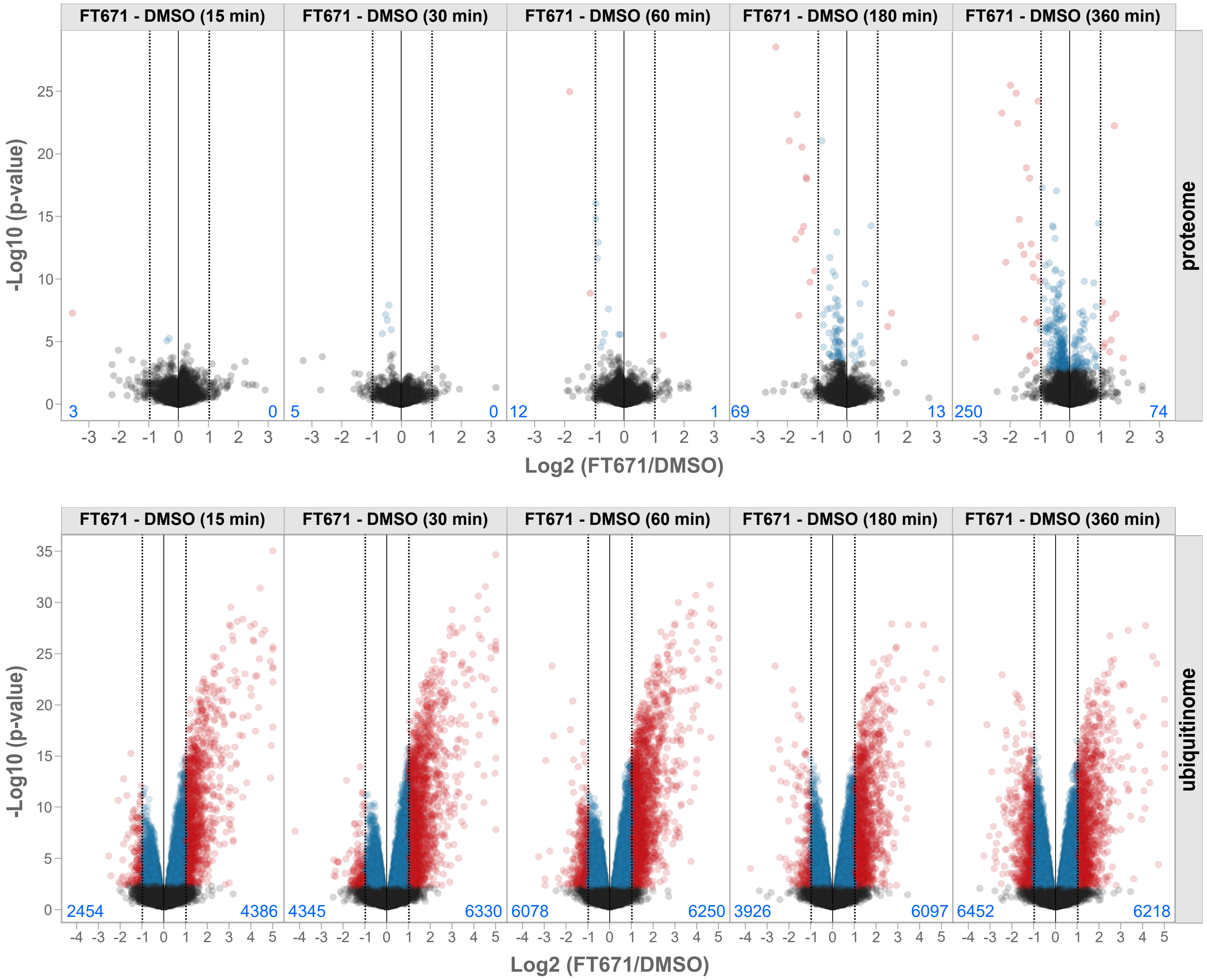

Supplementary Figure 3. USP7-dependent changes in the ubiquitinome and the proteome.

Volcano plot visualizations of Log2-transformed, averaged FT671/DMSO ratios versus their p-values (-Log10 transformed), as determined by quantitative DIA-MS analysis of proteins (upper panels) and ubiquitinated peptides (lower panels). Significantly-regulated proteins and K-GG peptides are colored (Log2 fold changes $-1 < x < 1$ in blue and $x < -1$ or $x > 1$ in red) and the number of significant down- and up-regulations at 5% FDR (LIMMA[1]) are indicated for each volcano plot. The data were filtered for 50% valid values across all conditions before statistical testing. 46,927 K-GG peptides and 10,569 proteins passed this filtering step.

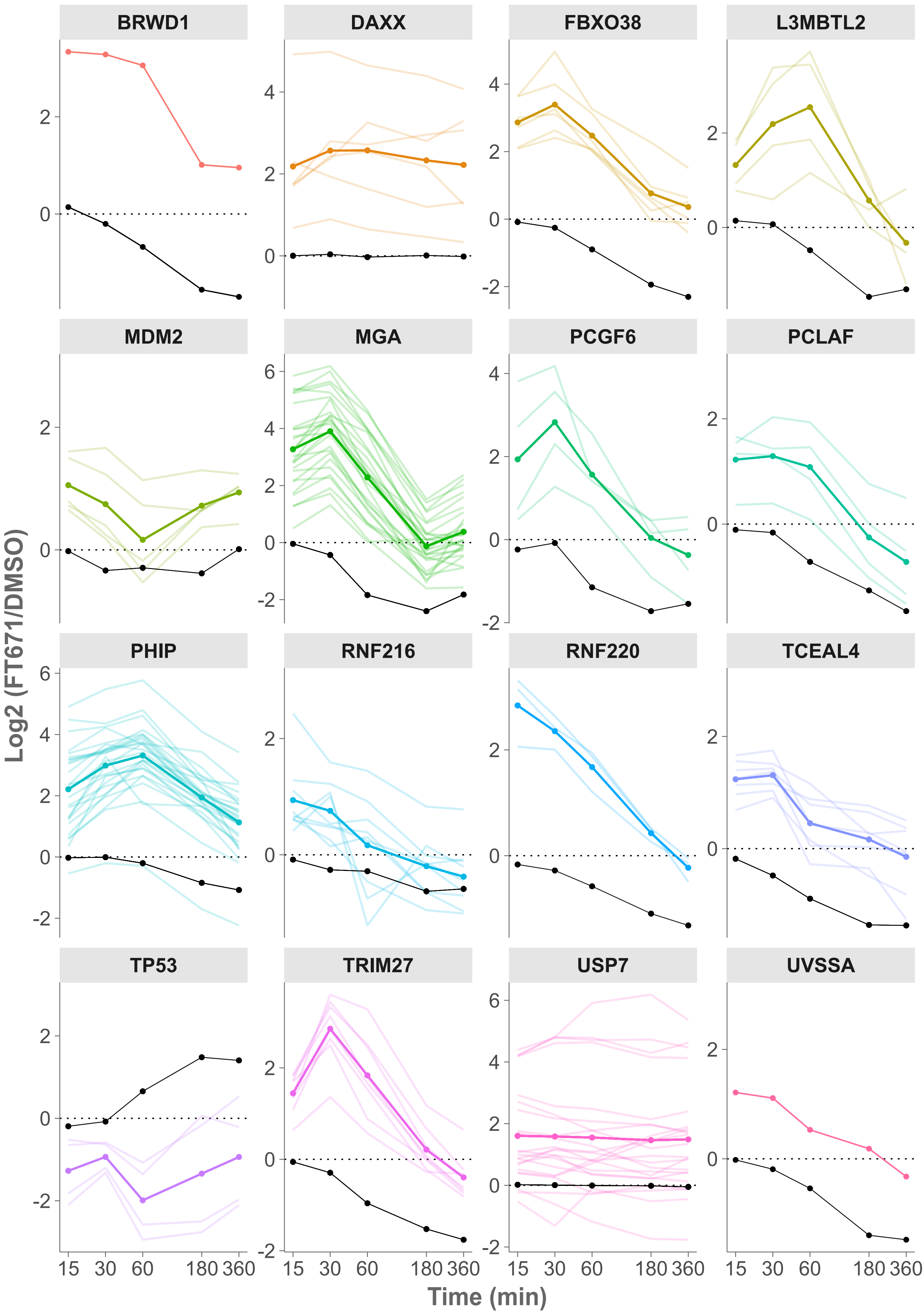
BRWD1
DAXX
FBXO38
L3MBTL2
MDM2
MGA
PCGF6
PCLAF
PHIP
RNF216
RNF220
TCEAL4
TP53
TRIM27
USP7
UVSSA
Log2 (FT671/DMSO)
Time (min)
15 30 60 180 360

Supplementary Figure 4. Modulation of protein ubiquitination and protein stability upon USP7 inhibition.

Profile plots of selected targets showing MS-based quantifications of the proteins (in black) and their corresponding ubiquitination sites (colored) that were significantly regulated upon FT671 treatment. Both average (solid lines) and individual (transparent lines) ubiquitinated peptide profiles are plotted for five time points. The y-axis depicts the fold change (Log2) of FT671 versus DMSO. Source data are provided as a Source Data file.

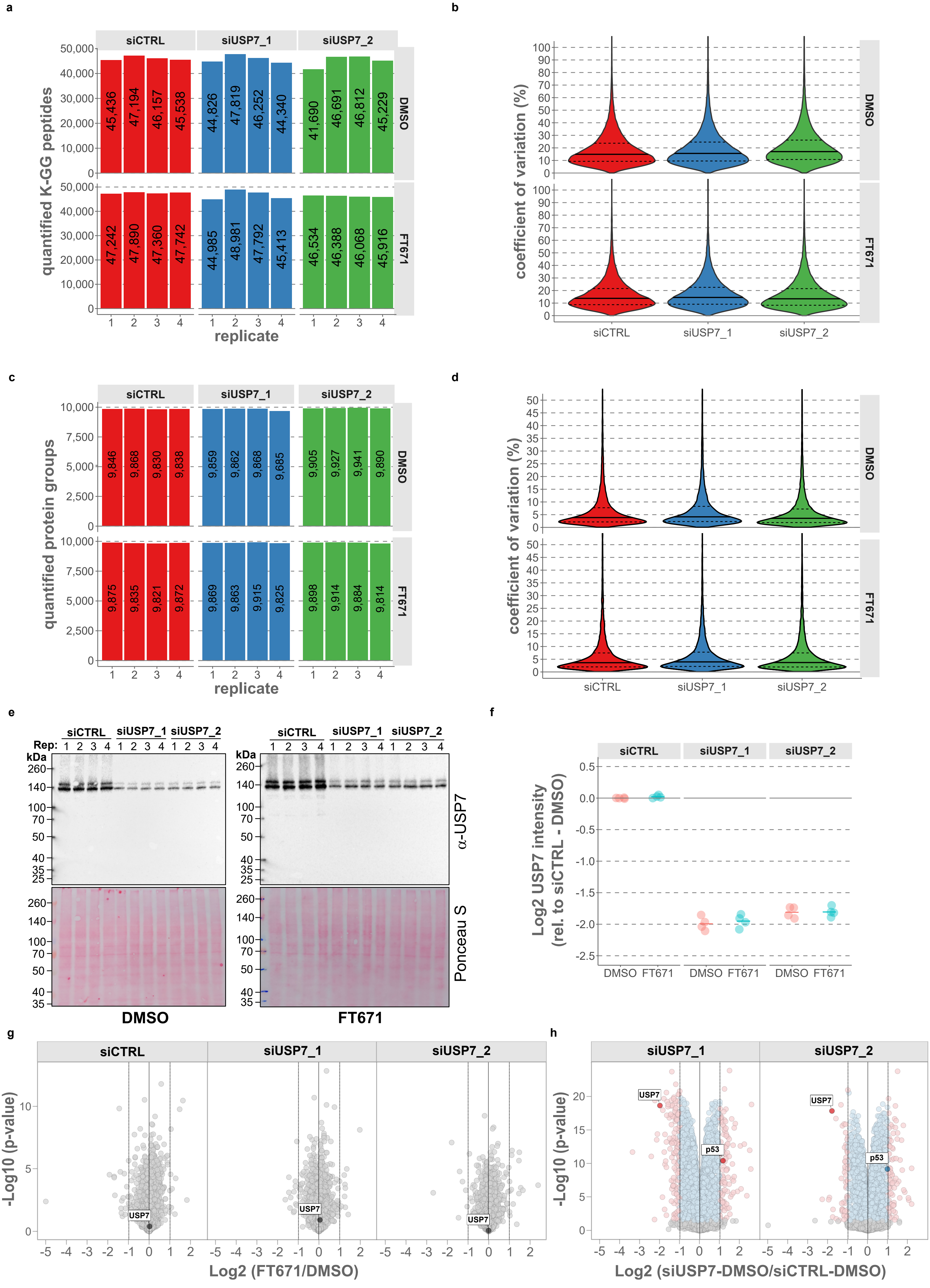
a
siCTRL
siUSP7_1
siUSP7_2
DMSO
FT671
quantified K-GG peptides
replicate
45,436
47,194
46,157
45,538
44,826
47,819
46,252
44,340
41,690
46,691
46,812
45,229
47,242
47,890
47,360
47,742
44,985
48,981
47,792
45,413
46,534
46,388
46,068
45,916
b
coefficient of variation (%)
c
quantified protein groups
9,846
9,868
9,830
9,838
9,859
9,862
9,868
9,685
9,905
9,927
9,941
9,890
9,875
9,835
9,821
9,872
9,869
9,863
9,915
9,825
9,898
9,914
9,884
9,814
d
e
Rep:
kDa
α-USP7
Ponceau S
DMSO
FT671
f
Log2 USP7 intensity
(rel. to siCTRL - DMSO)
g
-Log10 (p-value)
USP7
Log2 (FT671/DMSO)
h
p53
Log2 (siUSP7-DMSO/siCTRL-DMSO)

Supplementary Figure 5. siRNA-mediated knock-down of USP7 in HCT116 cells.

a Number of MS-quantified K-GG peptides for all replicates and treatment conditions using a 75 min DIA-MS method. Cells were transfected with a control siRNA or two different pools of siRNAs each targeting USP7 for 48 h and treated with DMSO or 10 µM FT671 for 5 minutes before harvesting. Four biological replicates were processed for each condition. **b** Violin plot showing coefficients of variation (CVs) distributions for K-GG peptides quantified in **a**. Continuous lines in the violin plots demarcate the median and dashed lines upper and lower quartiles. **c** Number of MS-quantified protein groups for all replicates and treatment conditions. An aliquot of the samples shown in **a** was used for whole proteome analysis by DIA-MS. The data were acquired using a 75 min DIA-MS method. **d** Violin plot showing coefficients of variation (CVs) distributions for protein groups quantified in **c**. Continuous lines in the violin plots demarcate the median and dashed lines upper and lower quartiles. **e** Western blot analysis of the samples used for ubiquitinome and proteome analysis (**a-d**). 50 µg of total protein/sample was resolved on SDS-PAGE, followed by transfer to nitrocellulose membranes and probing using a USP7-specific antibody. Uncropped plots in Source Data. **f** Dot plot showing MS-quantified intensities of USP7 after treatment with control or USP7-targeting siRNAs. The intensities were normalized (median subtraction) to the control condition (siCTRL, DMSO treated). Four biological replicates were processed for each condition. **g** Volcano plot visualizations of Log2-transformed, averaged FT671/DMSO protein level ratios versus their p-values (-Log10 transformed) after treatment with control or USP7-specific siRNAs for 48 h and treatment with DMSO or 10 µM FT671 for 5 minutes. USP7 is marked in all plots. The data were filtered for 50% valid values across conditions before statistical testing. Significantly regulated proteins (LIMMA[1], 5% FDR) are colored according to their Log2 fold change ($-1 < x < 1$ in blue and $x < -1$ or $x > 1$ in red). n= 4. **h** Volcano plots showing a comparison of all expressed proteins in control conditions (DMSO treated), with or without knock-down of USP7. Both p53 and USP7 are marked in all plots. The data were filtered for 50% valid values across conditions before statistical testing. Significantly regulated proteins (LIMMA[1], 5% FDR) are colored according to their Log2 fold change ($-1 < x < 1$ in blue and $x < -1$ or $x > 1$ in red) n=4. Source data are provided as a Source Data file.

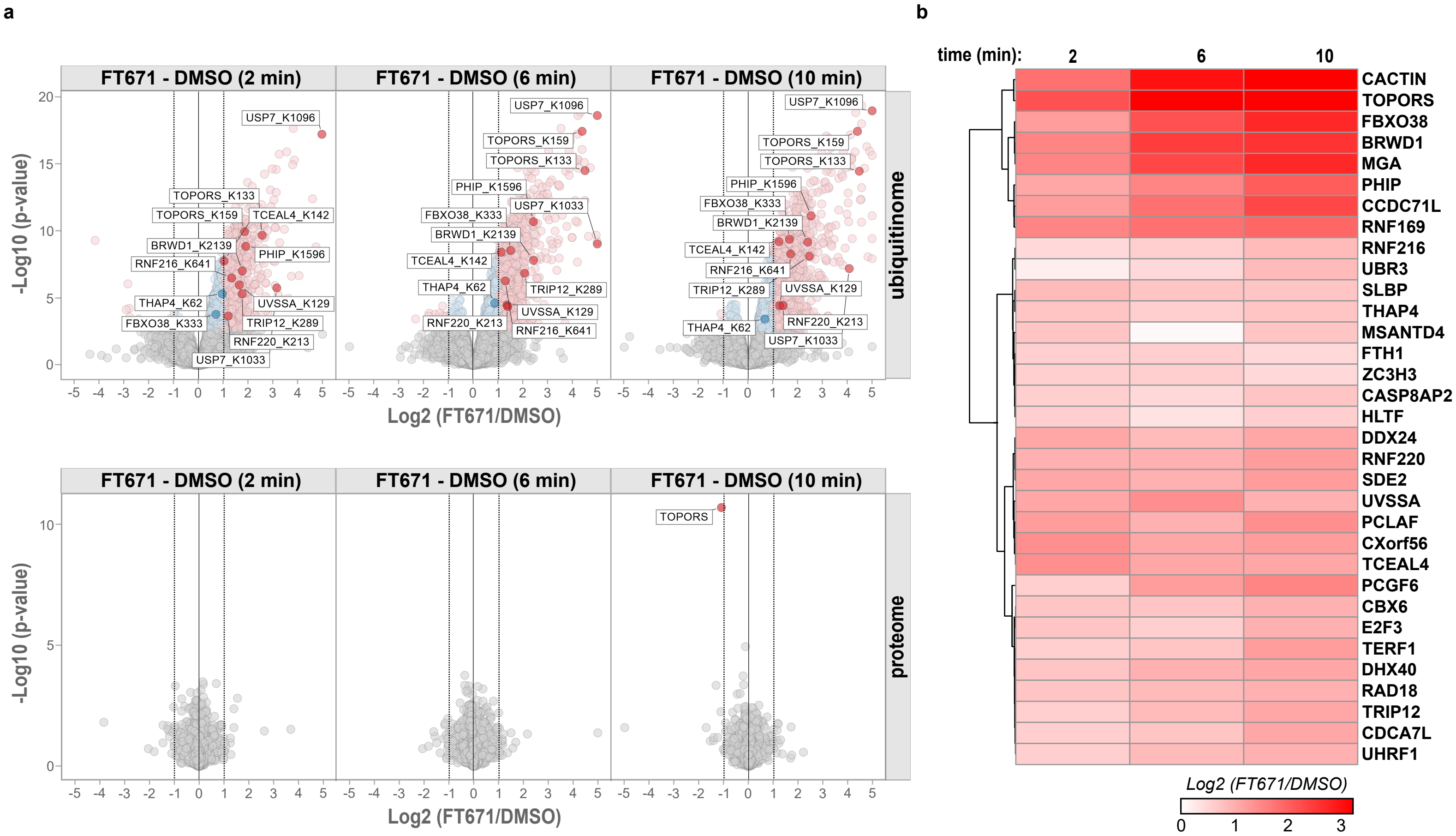

Supplementary Figure 6. FT671 time course experiment with short treatment times.

a HCT116 cells were treated with 10 µM FT671 for 2, 6 and 10 minutes. Volcano plots show Log2-transformed, average FT671/DMSO ratios versus their p-values (-Log10) for the ubiquitinome (upper panel) and the corresponding proteome (lower panel). Ubiquitinated peptides of selected targets are highlighted. Significantly-regulated K-GG peptides and proteins (LIMMA[1], 5% FDR) are colored according to their Log2 fold change ($-1 < x < 1$ in blue and $x < -1$ or $x > 1$ in red). **b** Heatmap of Log2-transformed ubiquitinated peptide fold changes (FT671/DMSO), of proteins that were significantly and more than 20% downregulated at later time points (15 min - 6 h, see Figure 4). Ubiquitinated peptide profiles (>2-fold upregulated) of the individual proteins were averaged.

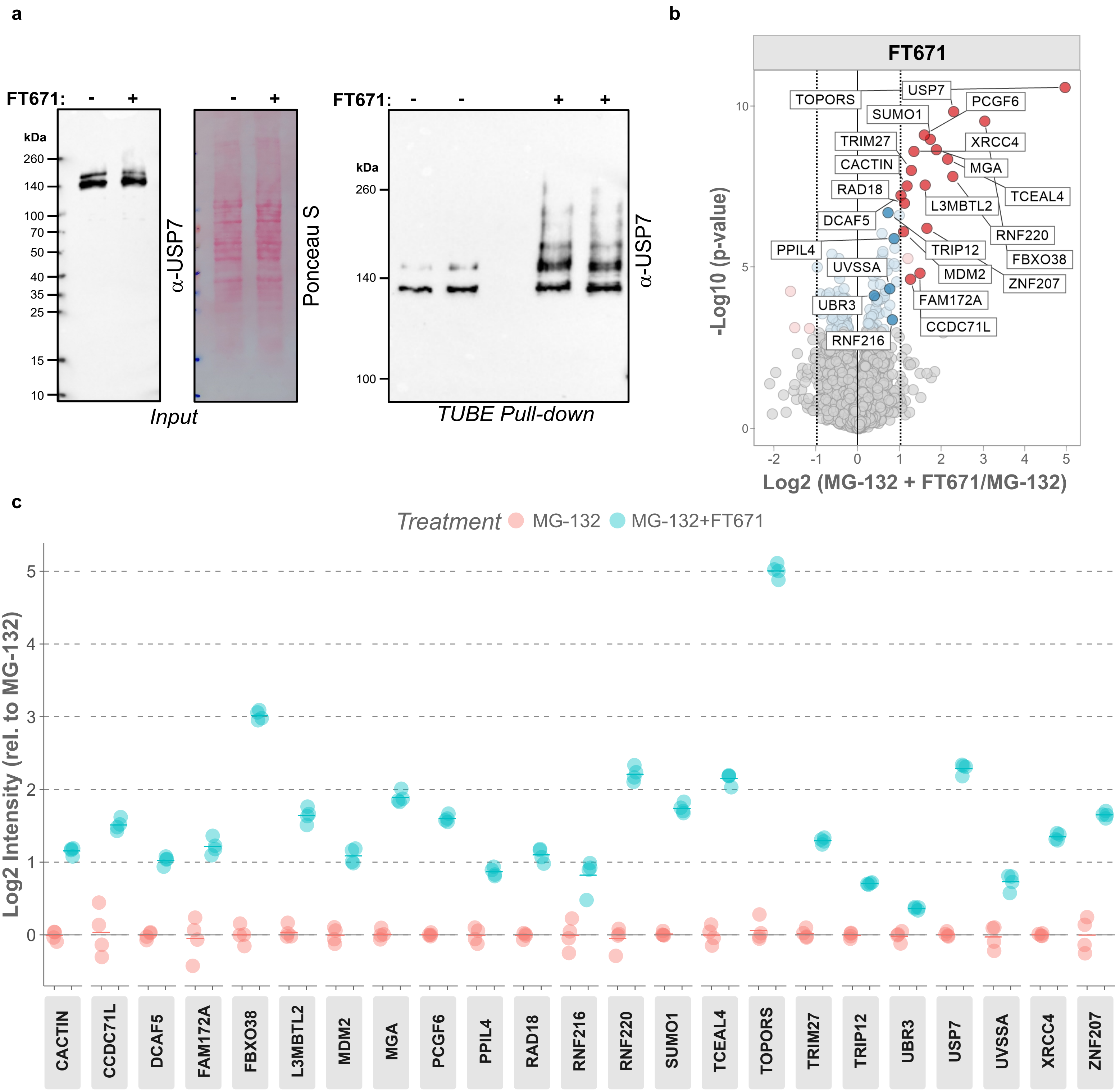
a
FT671: - +
kDa
260
140
100
70
50
40
35
25
15
10
α-USP7
Ponceau S
Input
FT671: - - + +
kDa
260
140
100
α-USP7
TUBE Pull-down
b
FT671
-Log10 (p-value)
Log2 (MG-132 + FT671/MG-132)
USP7
TOPORS
PCGF6
SUMO1
TRIM27
XRCC4
CACTIN
MGA
RAD18
TCEAL4
L3MBTL2
DCAF5
RNF220
PPIL4
TRIP12
FBXO38
UVSSA
MDM2
ZNF207
UBR3
FAM172A
CCDC71L
RNF216
c
Treatment MG-132 MG-132+FT671
Log2 Intensity (rel. to MG-132)
CACTIN
CCDC71L
DCAF5
FAM172A
FBXO38
L3MBTL2
MDM2
MGA
PCGF6
PPIL4
RAD18
RNF216
RNF220
SUMO1
TCEAL4
TOPORS
TRIM27
TRIP12
UBR3
USP7
UVSSA
XRCC4
ZNF207

Supplementary Figure 7. Detection of USP7 ubiquitination using ubiquitin binding domains.

a HCT116 cells were treated with 10 µM MG-132, alone or in combination with 10 µM FT671 for 15 minutes. Tandem ubiquitin binding domains (TUBE) coupled to agarose beads were used to enrich ubiquitinated proteins (2 mg of total protein as input, n=2), the eluate resolved on SDS-PAGE and the proteins transferred to a nitrocellulose membrane. USP7 was detected by chemiluminescence. The input is shown on the left, for which 50 µg of total lysate were loaded onto the gel. Uncropped plots in Source Data. **b** Using the pulldown approach described in **a**, ubiquitinated proteins were enriched and on-bead digested using trypsin, followed by LC-MS analysis. The volcano plot visualization shows averaged and Log2-transformed protein fold changes (FT671+MG-132/MG-132, x-axis) and p-values (-Log10 transformed) on the y-axis. Significantly regulated proteins (LIMMA[1], 5% FDR) are colored according to their Log2 fold change ($-1 < x < 1$ in blue and $x < -1$ or $x > 1$ in red). Selected examples of significantly scored proteins, for which ubiquitination sites were significantly induced in the K-GG proteome after 15 minutes of FT671 treatment (see Figure 4 and Supplementary Data 7) are highlighted. USP7 and Topors were among the most strongly enriched proteins. **c** Dot plot showing Log2-transformed protein intensities (each normalized to its control by median subtraction, i.e. MG-132 treatment) for the targets highlighted in **b**. Source data are provided as a Source Data file.

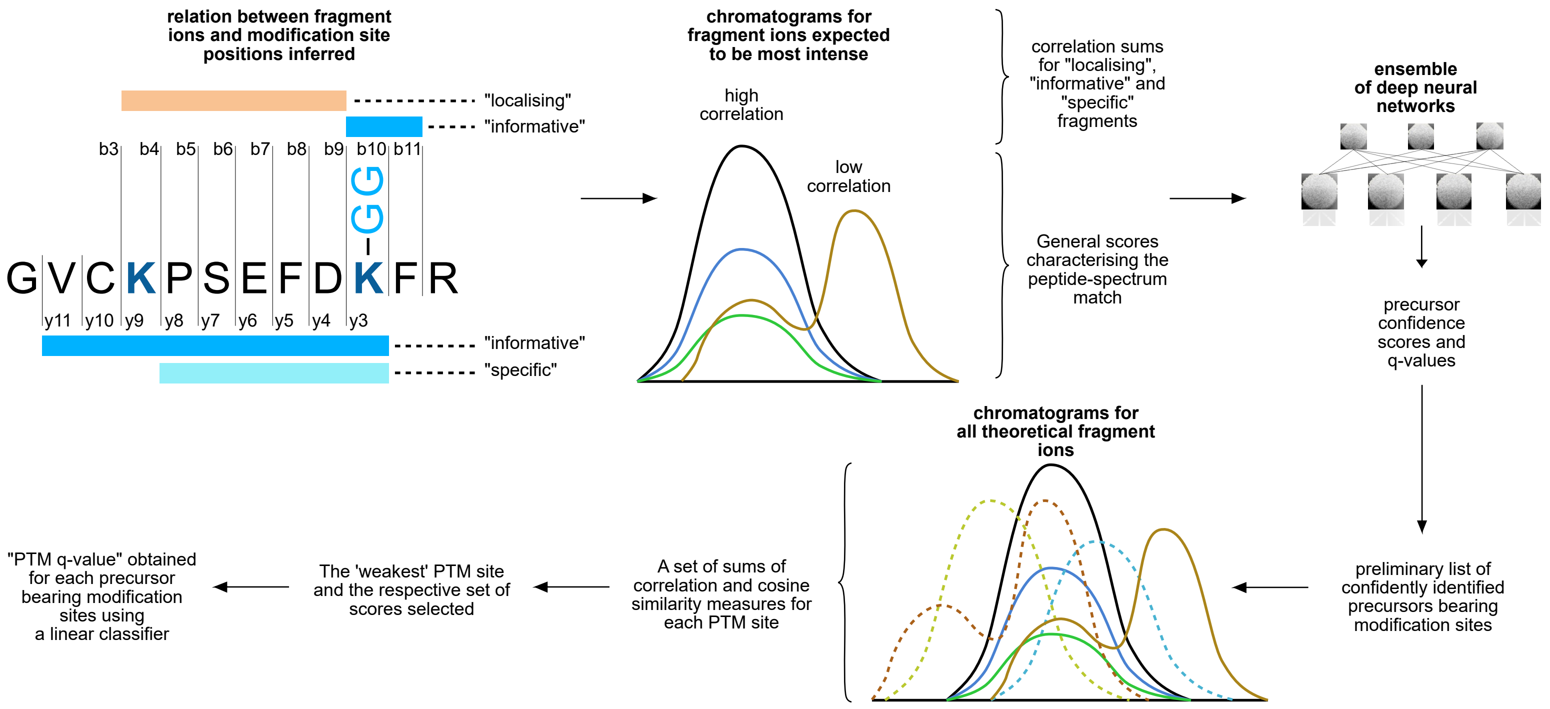

Supplementary Figure 8. PTM scoring module as implemented in DIA-NN.

DIA-NN calculates a ‘PTM q-value’ for each precursor, which reflects the identification confidence for the specific set of modifications present. First, three types of scores are calculated for each peptide-spectrum match (PSM), which are then used in downstream PSM confidence assessment. These scores are also reported in the final analysis output. The scores are calculated using extracted elution profiles of fragment ions that are predicted to have the most intense signals. The “informative” score is calculated using all fragment ions containing all the modified residues. The “localising” score - using all fragment ions that do not contain the modified residues, but do contain all other potential (unoccupied) modification sites, if any are present. Finally, the “specific” score - using all fragment ions that only contain the modified residues, but not any other potential modification sites. In each case, the sums of non-negative correlations of these fragments’ elution profiles with the designated ‘best’ fragment’s elution profile (as defined previously[24]) are calculated and used, among other scores, by the deep neural network classifier to assign confidence scores to the peptide-spectrum matches[24]. This allows to assemble a preliminary list of confidently identified precursors bearing modification sites. For each of these, chromatograms are extracted for all theoretical fragment ions. Then, for each of the modification sites, sums of cubes of correlation scores of all fragments containing the site are calculated. The site with the ‘weakest’ evidence is chosen and scored, using non-negative correlation and cosine similarity sums (regular as well as sums of cubes) across elution profiles of fragments that contain this site. These scores form the basis of confidence assessment for the PSM. To evaluate how significant these correlation/cosine sums are in comparison to the expected noise, the latter is modelled, for precursors with one or more modified residues, by ‘stripping’ the modifications from the precursor in silico, and calculating the respective correlation/cosine sums. A linear classifier is then trained, as described previously[24], to distinguish between true signals and noise, based on the differences between the respective scores for the target modified precursor and the stripped precursor, to generate a composite score reflecting the significance of the MS2-level evidence for the particular set of modifications. Calculated for both the target and in silico ‘stripped’ precursors, these composite scores allow to obtain q-values. MS1-level evidence is taken into account in a similar manner, using the correlation between the extracted MS1-level elution profile of the precursor ion with the elution profile of the ‘best’ fragment (as defined previously[24]), with the expected noise being modelled by calculating the correlation score for the extracted elution profile for the mass m/z - 1.003355, where m/z is the mass-to-charge ratio of the precursor, and 1.003355 is the ^{13}C - ^{12}C mass delta. Finally, q-values calculated at the MS2 and MS1 level are multiplied, to obtain the PTM q-value, which is used to filter out false matches.

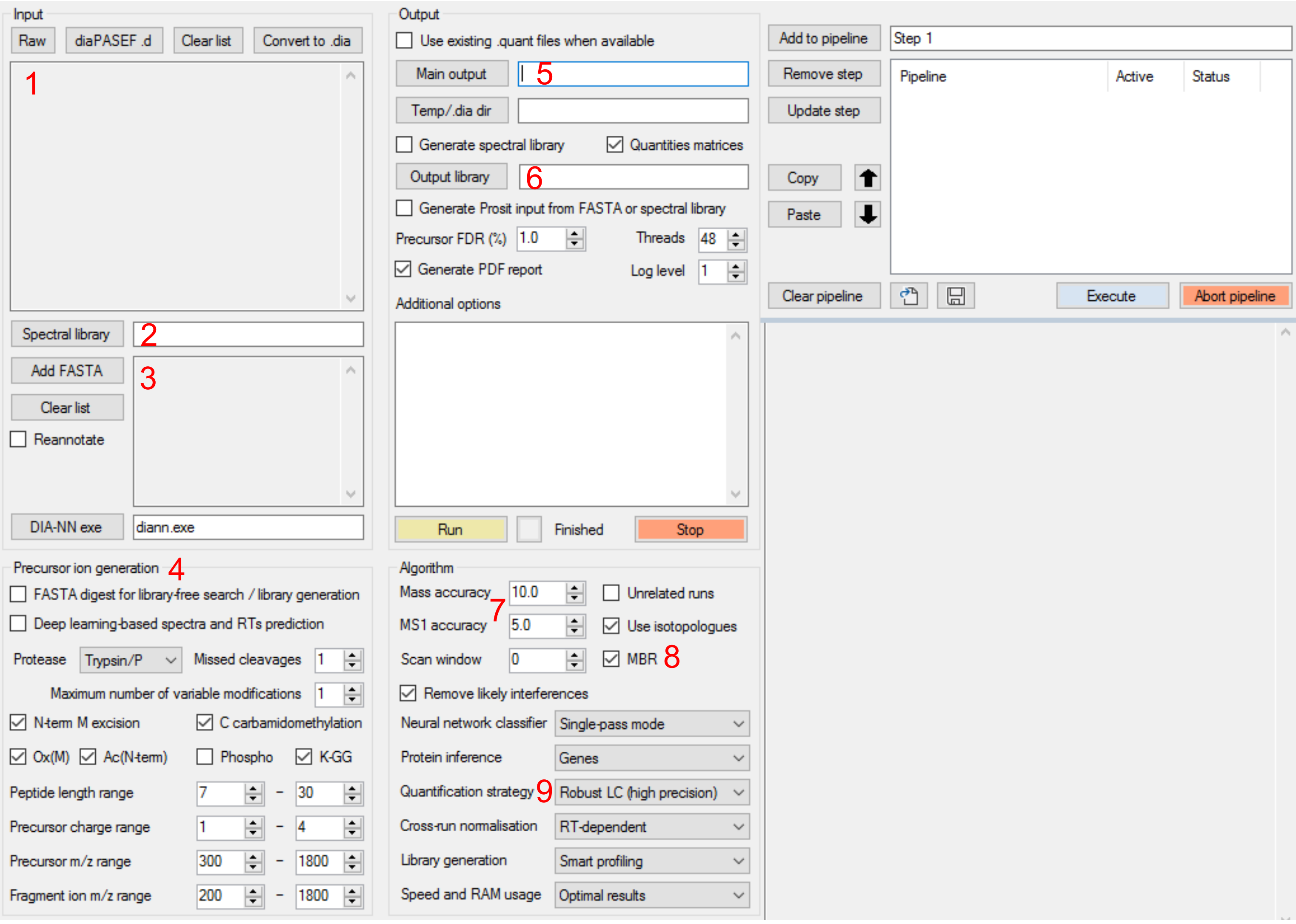

Supplementary Figure 9. Setup for library-free and spectral library-based searches in DIA-NN.

Graphical user interface of DIA-NN. (1) Specify raw files. (2) Specify a spectral library, only in case of spectral library-based searches. (3) Add FASTA file(s). (4) Enable both 'FASTA digest for library - free search/library generation' and 'Deep learning-based spectra and RTs prediction' in case of library-free searches. Additional parameters such as precursor charges, peptide length, allowed number of missed cleavages and variable modification can be set. For this study, one missed cleavage was allowed for both ubiquitinome and proteome analyses. For ubiquitinomics, two variable modifications per peptide were allowed (with M(Ox), K-GG and N-Ac). For proteome processings, one variable modification per peptide was enabled (with the variable modifications M(Ox) and N(Ac)). (5) Specify directory for the report output file, which contains all relevant information for downstream statistical testing, such as precursor/protein group intensities. (6) Output directory for the spectral library, if desired. (7) MS1/MS2 mass accuracies, in this work set to 5 and 10 ppm, respectively. (8) Match-between-runs (MBR), activated by default. Instructs DIA-NN to create a spectral library consisting of all identifications that are extracted from the specified raw files. This refined spectral library is then used for data processing in a second step. This strategy helps to i) reduce missing values ii) increase identifications and iii) increase quantitative precision. (9) Quantification strategy set to Robust LC (high precision). Recommended setting for achieving high quantitative precision when the LC setup is stable.

Supplementary Note 1: Identification confidence for K-GG peptides with DDA and DIA

To experimentally validate the identification confidence specifically for K-GG peptides, we mixed different amounts of tryptic *E. coli* digest (50/100/200/400 ng) with K-GG peptides enriched from MM.1S cells. In this setup, each K-GG peptide call from *E. coli* corresponds to a false positive identification. We acquired the data both in DDA and in DIA mode and processed the DDA raw data with MaxQuant (MBR enabled). We analyzed the DIA data by both Spectronaut and DIA-NN (library-free search mode). DDA identified about 7,800 human K-GG peptide precursors with 50 ng of *E.coli* digest added, and 5,800 precursors when adding 400 ng. Spectronaut quantified about twice the number at each tested human/*E.coli* mixing ratio. DIA-NN instead identified between 28,800 and 26,300 human K-GG peptides at the different mixing ratios. The *E.coli*/human K-GG peptide ratio was comparably low for both Spectronaut and DIA-NN (between 0.05-0.10%) and lower than for MaxQuant (Supplementary Figure 10).

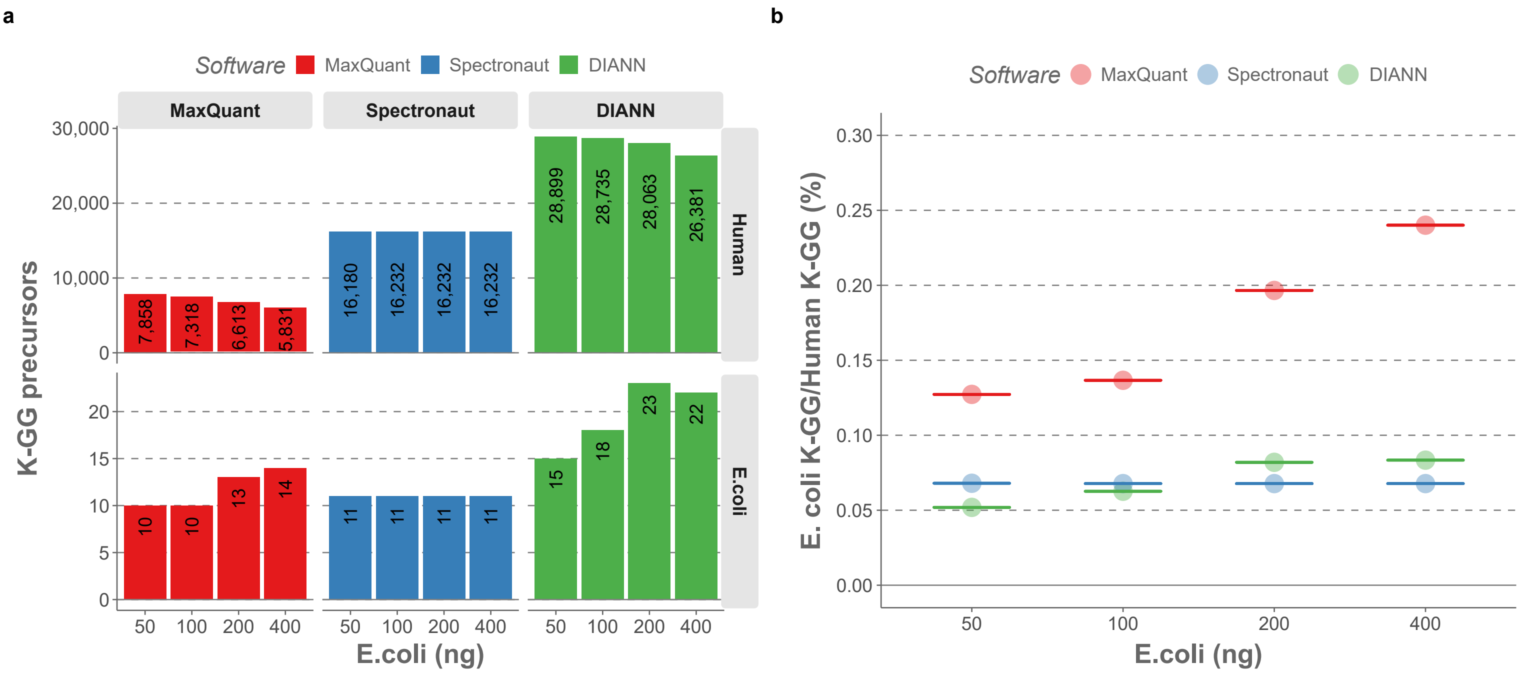

Supplementary Figure 10. Identification confidence for K-GG peptides with DDA and DIA.

Different amounts of *E. coli* tryptic digest (50, 100, 200, 400 ng) were mixed with human K-GG peptides obtained from bortezomib-treated MM.1S cells (1 mg of protein input) and the data was acquired with either DDA or DIA (75 min LC-MS methods). The DIA data was processed with either DIA-NN or Spectronaut, both operated in library-free mode. The DDA data was processed with MaxQuant (with MBR enabled). **a** Number of identified human and *E.coli* K-GG precursors with DDA and DIA. DIA-NN quantified about 75% more K-GG precursors than Spectronaut and about 4 times more than MaxQuant. **b** Ratio of *E. coli* versus human K-GG precursors (%) at different spike-in amounts of *E.coli* tryptic digest. Source data are provided as a Source Data file.

Supplementary Note 2: Quantification accuracy and precision for ubiquitinomics with DDA and DIA

To benchmark the dynamic range and the quantitative accuracy of our DIA method, we spiked 100 synthetic K-GG peptides into 200 ng of tryptic yeast digest in different amounts (100/10/1/0.1 fmol/peptide) and measured technical quadruplicates for each of DDA and DIA. Both scan modes identified all hundred synthetic K-GG peptides in at least one sample. However, DDA quantified only 60 synthetic peptides in all four replicates at the lowest spike-in concentration. DIA instead quantified 99 of them at a substantially higher quantification precision. DIA-NN also improved quantification performance, especially for the 1:1000 ratio (Supplementary Figure 11).

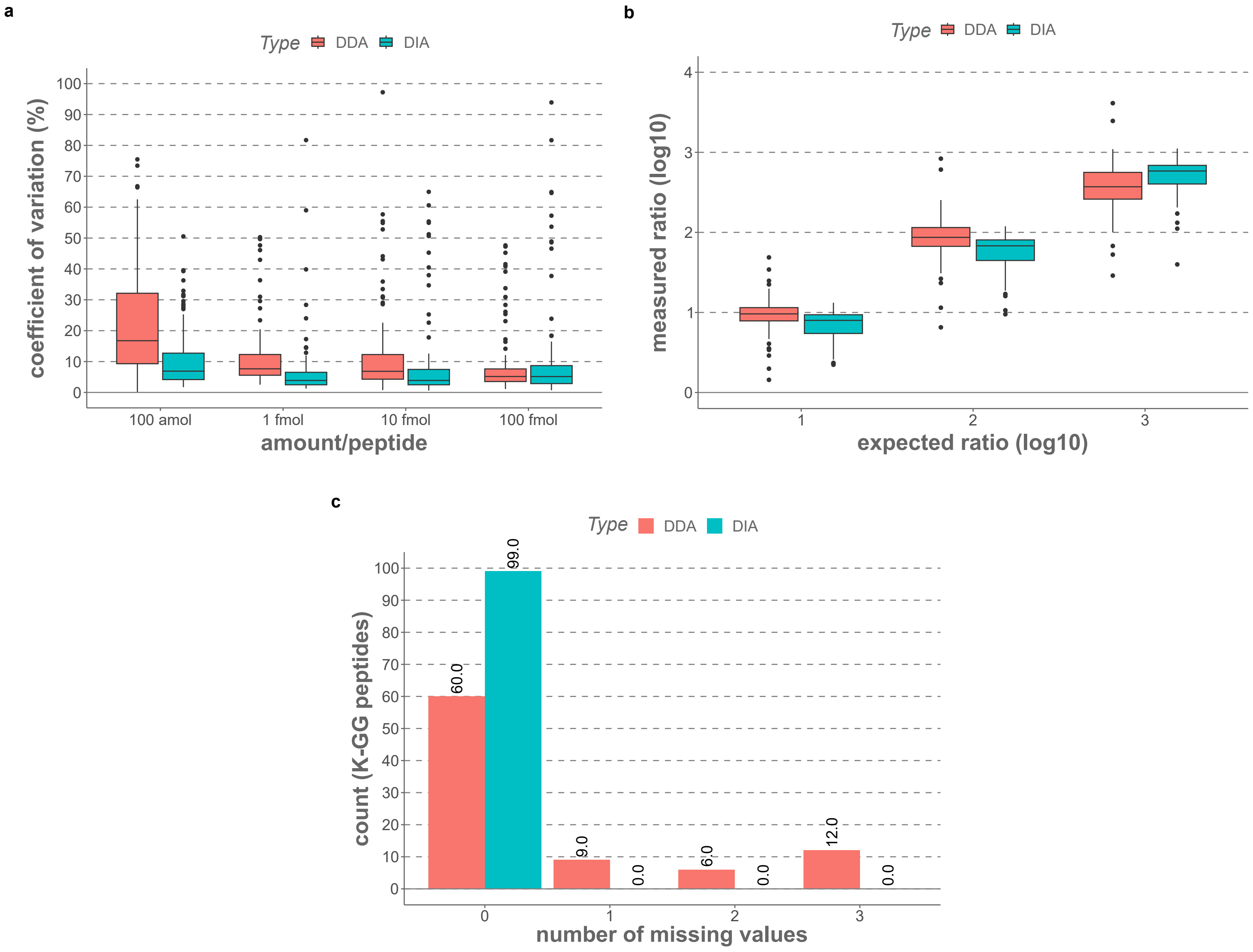

Supplementary Figure 11. Benchmarking DIA ubiquitinomics.

a 200 ng of yeast tryptic digest was mixed with 100 synthetic K-GG peptides at different amounts (0.1/1/10/1000 fmol/peptide) and the data was acquired using 75 min DDA or DIA methods. The raw files were processed with either MaxQuant or DIA-NN, for DDA and DIA, respectively. The coefficients of variation of the 100 spike-in peptides were computed on raw intensities for both scan modes (n= 4). **b** Measured vs expected fold changes (1:10, 1:100, 1:1000, log10) for the synthetic peptides in DDA/DIA mode. **c** Number of quantified synthetic peptides vs number of missing values for DDA and DIA in quadruplicate measurements with 100 amol/peptide added. 99 K-GG peptides were identified in all four replicates with DIA (60 with DDA). Source data are provided as a Source Data file.

Supplementary references

1 Ritchie, M. E. *et al.* limma powers differential expression analyses for RNA-sequencing and microarray studies. *Nucleic Acids Res.* **43**, e47 (2015).

2 Demichev, V., Messner, C. B., Vernardis, S. I., Lilley, K. S. & Ralser, M. DIA-NN: neural networks and interference correction enable deep proteome coverage in high throughput. *Nat. Methods* **17**, 41–44 (2020).
