# Supplementary material for: Time-resolved in vivo ubiquitinome profiling by DIA-MS reveals USP7 targets on a proteome-wide scale
Source: Nat Commun. 2021 Sep 13;12:5399. doi: 10.1038/s41467-021-25454-1 (PMC8438043; doi:10.1038/s41467-021-25454-1)
Supplement: Supplementary file 3 — Description of Additional Supplementary Files [file 41467_2021_25454_MOESM3_ESM.docx]

**File Name: Supplementary Data 1**

Description: MaxQuant outputs (modification specific peptides and K-GlyGly sites) of sodium deoxycholate (SDC)- and urea-lysis protocols

**File Name: Supplementary Data 2**

Description: MaxQuant outputs (modification specific peptides and K-GlyGly sites) for different protein inputs from Jurkat cells.

**File Name: Supplementary Data 3**

Description: MaxQuant outputs (modification specific peptides and K-GlyGly sites) for the sodium deoxycholate (SDC) lysis benchmark experiment.

**File Name: Supplementary Data 4**

Description: MS and LC parameters for the different LC-MS methods used.

**File Name: Supplementary Data 5**

Description: List of quantified peptides by DIA-MS from different proteins inputs of MG-132-treated HCT116 cells.

**File Name: Supplementary Data 6**

Description: List of all quantified K-GG peptides with the different LC-MS methods.

**File Name: Supplementary Data 7**

Description: Quantified K-GG peptides and proteins in a time course experiment with FT671.

**File Name: Supplementary Data 8**

Description: List of K-GG peptides and proteins of putative USP7 substrates.

**File Name: Supplementary Data 9**

Description: List of K-GG peptides and proteins quantified upon knock-down of USP7 and treatment with FT671.

**File Name: Supplementary Data 10**

Description: K-GG remnant peptides quantified in a high-resolution FT671 time course experiment.

**File Name: Supplementary Data 11**

Description: Quantified K-GG peptides upon treatment of HCT116 cells with four structurally distinct USP7 inhibitors.

**File Name: Source Data**

Description: Raw data underlying display data
