# Supplementary material for: Time-resolved in vivo ubiquitinome profiling by DIA-MS reveals USP7 targets on a proteome-wide scale
Source: Nat Commun. 2021 Sep 13;12:5399. doi: 10.1038/s41467-021-25454-1 (PMC8438043; doi:10.1038/s41467-021-25454-1)
Supplement: Supplementary file 15 — Reporting Summary [file 41467_2021_25454_MOESM15_ESM.pdf]

nature portfolio

Corresponding author(s): Martin Steger
Last updated by author(s): Aug 6, 2021

Reporting Summary

Nature Portfolio wishes to improve the reproducibility of the work that we publish. This form provides structure for consistency and transparency in reporting. For further information on Nature Portfolio policies, see our Editorial Policies and the Editorial Policy Checklist.

Statistics

For all statistical analyses, confirm that the following items are present in the figure legend, table legend, main text, or Methods section.

n/a	Confirmed	
☐	☒	The exact sample size (*n*) for each experimental group/condition, given as a discrete number and unit of measurement
☐	☒	A statement on whether measurements were taken from distinct samples or whether the same sample was measured repeatedly
☐	☒	The statistical test(s) used AND whether they are one- or two-sided<br>*Only common tests should be described solely by name; describe more complex techniques in the Methods section.*
☒	☐	A description of all covariates tested
☐	☒	A description of any assumptions or corrections, such as tests of normality and adjustment for multiple comparisons
☐	☒	A full description of the statistical parameters including central tendency (e.g. means) or other basic estimates (e.g. regression coefficient) AND variation (e.g. standard deviation) or associated estimates of uncertainty (e.g. confidence intervals)
☐	☒	For null hypothesis testing, the test statistic (e.g. *F*, *t*, *r*) with confidence intervals, effect sizes, degrees of freedom and *P* value noted<br>*Give P values as exact values whenever suitable.*
☒	☐	For Bayesian analysis, information on the choice of priors and Markov chain Monte Carlo settings
☐	☒	For hierarchical and complex designs, identification of the appropriate level for tests and full reporting of outcomes
☐	☒	Estimates of effect sizes (e.g. Cohen's *d*, Pearson's *r*), indicating how they were calculated

Our web collection on statistics for biologists contains articles on many of the points above.

Software and code

Policy information about availability of computer code

Data collection	The following software was used: MaxQuant (version 1.6.17.0), Spectronaut (version 14.10.201222.47784), DIANN (version 1.8), Thermo Xcalibur (version 4.2.47), LTQ Tune plus (version 2.11 QF1 Built 3006)
Data analysis	The following software/codes were used: MaxQuant (version 1.6.17.0), Cytoscape (version 3.7.2), Spectronaut (version 14.10.201222.47784), DIANN (version 1.8), RStudio (version 3.6.1), Perseus (version 1.6.14.0), Spotfire (version 7.12.0), MaxLFQ (as implemented in DIA-NN), ID Picker (J. Proteome Res. 6, 3549–3557 (2007))

For manuscripts utilizing custom algorithms or software that are central to the research but not yet described in published literature, software must be made available to editors and reviewers. We strongly encourage code deposition in a community repository (e.g. GitHub). See the Nature Portfolio guidelines for submitting code & software for further information.

Data

Policy information about availability of data

All manuscripts must include a data availability statement. This statement should provide the following information, where applicable:

- Accession codes, unique identifiers, or web links for publicly available datasets
- A description of any restrictions on data availability
- For clinical datasets or third party data, please ensure that the statement adheres to our policy

The mass spectrometry raw data and the corresponding preocessing reports have been deposited to the ProteomeXchange Consortium (http://proteomecentral.proteomexchange.org) via the PRIDE partner repository with the dataset identifier PXD023889. Previously published data were also used in various benchmark experiments (repositories with identifiers PXD019854 and PXD006201). DIA-NN is freely available for download at https://github.com/vdemichev/diann. The links of raw files to their respective experiments are available in the Source Data file. Annotation information of proteins (gene annotation (GO) terms) were

obtained from UniProt (https://www.uniprot.org/).Source data are provided with this paper.

Field-specific reporting

Please select the one below that is the best fit for your research. If you are not sure, read the appropriate sections before making your selection.

- [x] Life sciences
- [ ] Behavioural & social sciences
- [ ] Ecological, evolutionary & environmental sciences

For a reference copy of the document with all sections, see nature.com/documents/nr-reporting-summary-flat.pdf

Life sciences study design

All studies must disclose on these points even when the disclosure is negative.

Sample size	No sample size calculation was done. We acquired 4 replicate samples/condition (independent cell treatments/sample preparation) for all biological and protocol benchmark experiments. This number is sufficient for robust statistical analyses. For results reported in Supplementary Fig. 1a, we used three replicates only. This was sufficient to determining the effect of temperature on lysine modification. For the experiment shown in Supplementary Fig. 10, only one replicate/condition was acquired. As the purpose of this experiment was to compare different software in terms of identification confidence (no quantification information used), this is sufficient. When the purpose of the experiment was to compare MS Methods or LC gradients, the same sample was injected multiple times into the mass spectrometer (analytical replicates). This is necessary to avoid unwanted biases introduced by e.g. sample preparation and hence to guarantee comparability
Data exclusions	No data was excluded
Replication	Four independent treatments (individual cell culture plates) for each time point were analyzed for all USP7 inhibitor experiments (biological replicates). For all SDC/urea buffer benchmark experiments, four individual samples were used for K-GG peptide enrichment (workflow replicates). For the direct comparison of DDA/DIA, the same sample was injected four times for each acquisition mode using block randomization (analytical replicates). For USP7 knockdown experiments, four biological replicates were analyzed using Western blotting. For the USP7 WB shown in Supplementary Fig. 6, two workflow replicates (individual enrichment of ubiquitinated proteins using ubiquitin domains) were performed. Replication experiments (e.g. USP7 inhibitor treatment with different times) were successful. Reproducibility between replicates was confirmed by Pearson correlation coefficients, by coefficients of variation and/or by principal component analysis (PCA)
Randomization	All MS measurements were block randomized (e.g. control-replicate1, treatment1-replicate1, treatment2-replicate1, control-replicate2,...or DDA-rep1,DIA_rep1, DDA-rep2, DIA-rep2,...)
Blinding	No blinding was intentionally applied. However, each sample was assigned a number (without indication of the treatment) and the lab technicians were thus not aware of the treatments. Moreover, all data was collected by mass spectrometers and therefore not biased by the operators.

Reporting for specific materials, systems and methods

We require information from authors about some types of materials, experimental systems and methods used in many studies. Here, indicate whether each material, system or method listed is relevant to your study. If you are not sure if a list item applies to your research, read the appropriate section before selecting a response.

Materials & experimental systems

n/a	Involved in the study	
☐	☒	Antibodies
☐	☒	Eukaryotic cell lines
☒	☐	Palaeontology and archaeology
☒	☐	Animals and other organisms
☒	☐	Human research participants
☒	☐	Clinical data
☒	☐	Dual use research of concern

Methods

n/a	Involved in the study	
☒	☐	ChIP-seq
☒	☐	Flow cytometry
☒	☐	MRI-based neuroimaging

Antibodies

Antibodies used	USP7 antibody (Cell signaling technology, #4833, 1:1000), PTMScan Ubiquitin Remnant Motif (K-E-GG) Kit, for enriching K-GG peptides, supplied as agarose conjugate (Cell signaling technology, #5562), anti-rabbit HRP-linked antibody (Rockland (611-1302), 1:10000)
Validation	K-GG remnant antibodies (CST, 5562): validation performed by detecting K-GG remnant peptides by mass spectrometry.<br>USP7 antibody: https://www.cellsignal.de/products/primary-antibodies/hausp-d17c6-xp-rabbit-mab/4833?site-search-type=Products&N=4294956287&Ntt=usp7&fromPage=plp

Eukaryotic cell lines

Policy information about cell lines

Cell line source(s)	MM1.S, HCT116, Jurkat 6.1 (ATTC, https://www.atcc.org/). Data from previously published studies with U2OS and HEK293 cells was used for benchmarking experiments (see 'data availablity' section and the manuscript for details). These cells were not cultured for this study.
Authentication	No authentication was performed
Mycoplasma contamination	All cell lines used were regularly tested and Mycoplasma negative
Commonly misidentified lines (See ICLAC register)	None
